# Do MCHC, MPV, and Procalcitonin Levels Determine Prognosis in Acute Coronary Syndrome?

**DOI:** 10.1155/2019/6721279

**Published:** 2019-07-18

**Authors:** Serhat Karaman, Abuzer Coskun

**Affiliations:** ^1^Department of Emergency Medicine, Gaziosmanpasa University, Faculty of Medicine, Tokat, Turkey; ^2^Department of Emergency Medicine, Sivas State Hospital, Sivas, Turkey

## Abstract

**Aim:**

Acute coronary syndrome (ACS) continues to be the main cause of mortality and morbidity globally. The aim was to assess serum procalcitonin (PCT), mean corpuscular hemoglobin concentration (MCHC) and mean platelet volume (MPV) levels in terms of complications after myocardial infarctus, triple vein coronary artery disease (TVCAD), and mortality prediction.

**Material and Method:**

This cross-sectional cohort study included 200 patients with ACS attending the emergency department of our hospital with chest pain and admitted to the cardiology clinic from January 2014 to December 2016. Patients were divided into 4 groups as inferior group, anterior group, NSTEMI group, and UA group according to diagnosis. These groups were compared in terms of complications occurring after MI, TVCAD, and mortality rates.

**Results:**

There were significant differences in terms of complications forming after ACS, TVCAD, and mortality. The inferior subgroup had high PCT and MCHC levels and was found to have more complications developing and mortality compared to other groups. Patients with high PCT and MPV values were identified to have higher mortality and TVCAD. In the anterior subgroup, ischemic heart failure was higher compared to the other groups. In the interior, anterior, and non-ST elevated myocardial infarctus (NSTEMI) groups, the 0-, 6-, and 12-hour cTnI values were significantly higher compared to the UA group, while the anterior group had a significantly higher 12-hour cTnI value compared to the NSTEMI group. Correlation analysis for PCT, MCHC, and MPV with complications developing after MI, mortality, and TVCAD found positive and statistically significant correlations.

**Conclusion:**

High PCT, MCHC, and MPV levels in acute coronary syndrome may be beneficial predictive values in terms of complications that may develop, TVCAD, and mortality.

## 1. Introduction

In spite of developing medical technologies and treatments, globally ACS continues to be the main cause of mortality and morbidity [1, 2]. Early diagnosis and determination of prognosis are important for treatment and surveillance strategies.

Cardiac troponin (cTn) is a sensitive and specific marker for heart muscle injury.

But in recent times, there are many studies about new cardiac markers sensitive and specific for heart muscle injury and one of these is procalcitonin (PCT). Inflammation plays a role in the pathophysiology of atherosclerosis. PCT is defined as an inflammation marker. The original product of the Calc-1 gene is a preprocalcitonin 141-amino acid chain which binds to the N terminus of endoplasmic reticulum in C-cells in the thyroid gland and degrades with an endopeptidase to oxidize to PCT. Later PCT itself is cleaved by a convertase enzyme into calcitonin, catacalcin, and a protein remnant [3]. PCT concentration cannot be identified or is low in healthy individuals [4]. There are only a few studies about the effect of PCT on cardiovascular prognosis of patients with ACS diagnosis [5]. Recent studies have reported PCT levels are correlated with the degree of atherosclerosis in ACS patients [6–8].

Mean platelet volume (MPV) is a simple and reliable platelet measure index correlated with the functional status of platelets. Some studies in the literature have revealed MPV is an independent parameter for known risk factors for recurrent MI such as hypertension, dyslipidemia, increased fibrinogen, and white cell count and high MPV levels were identified with atherothrombosis [9, 10].

Anemia is known to be associated with poor prognosis in patients with ACS diagnosis. In patients with heart failure, an abnormal fall in hemoglobin content of erythrocytes called “relative hypochromia” was shown to be associated with more advanced left ventricle failure. The prognostic value of low mean corpuscular hemoglobin concentration (MCHC) is important especially for those with sufficient hemoglobin levels and abnormal MCHC values has additional prognostic importance [11, 12].

In our study, we aimed to assess PCT, cTn, MCHC, and MPV among STEMI, NSTEMI, and UA groups in terms of complications occurring after ACS, TVCAD, and mortality.

## 2. Material–Methods

### 2.1. Study Design and Population

The results of 200 patients (126 male, 74 female, mean age 63.53±9.44 years, and range 33-82 years) attending Sivas Numune Hospital, Emergency Department, from January 2014 to December 2016 with chest pain and admitted to the cardiology clinic with ACS preliminary diagnosis were prospectively obtained. Patients with no blood sugar, PCT, biochemistry, and hemogram were examined within the first 24 hours, and those without angiography or echocardiography performed after admission were excluded from the study. Patients were divided into 4 groups of 50 people. These were the inferior group (inferior AMI, inferolateral AMI, inferoposterior AMI, and right ventricle MI), anterior group (septal AMI, anterior AMI, lateral AMI, high lateral AMI, and widespread anterior AMI), and NSTEMI group and the fourth group comprised patients with high-risk UA diagnosis according to the Braunwald classification [13]. These four groups were researched in terms of age, sex, cTn, blood sugar, PCT, MCHG, MPV, and Gensini score. Troponin of patients was measured in blood samples taken on attendance at the emergency department, 6 hours and 12 hours later, and recorded as troponins I, II, and III.

Patients who had chest pain and/or discomfort lasting at least 30 minutes and ECG with STEMI according to 2013 ACCF/AHA guidelines were included in the study [14]. UA/NSTEMI was defined according to the criteria of the 2014 AHA/ACC Guideline for the Management of Patients with NSTE-ACS. All of the patients were checked with Transthoracic Echocardiography (TTE) to look for whether focal wall motion abnormalities were present or not. A Philips Epiq 7 Ultrasound Machine was used for TTE in this study.

All patients provided written informed consent and the study was approved by Cumhuriyet University, Medical School Ethical Committee.

The study was completed in accordance with the Declaration of Helsinki for Human Research and was approved by the institutional review board. Demographic, clinical, and laboratory data from the date of presenting to the ED due to ACS, including the PCT, MCHC, MPV, and cTnI levels, were assessed using a review of the hospital's medical records. MCHC and MPV were measured using a Beckman Coulter Automated CBC Analyzer (Beckman Coulter, Inc., Fullerton, CA, USA). The normal reference interval for MCHC and MPV was 30-36 mg/dL and 5.9-12fL, respectively.

### 2.2. Cardiac Biomarker Analysis

Venous blood samples from the antecubital veins of patients were obtained to measure serum levels of troponin I. Troponin I STATE lecsys and Cobas e 411 Hitachi Roche analyzers were used to measure troponin I levels. Patients had troponin levels measured at 0, 6, and 12 hours after attending the emergency department.

### 2.3. Procalcitonin

Procalcitonin was studied within 20-30 minutes with a Mini Vidas brand device. Results accepted 0-0.05 ng/mL as the normal interval, with high values accepted above this limit.

### 2.4. Angiographic Analysis

Angiographic evaluations were done by two experienced cardiologists, who were blinded to the study. Discrepancies were solved by consensus. The extent and severity of ACS were assessed with the Gensini score [15].

### 2.5. Statistical Analysis

The data obtained from this study was analyzed with SPSS 20 software package. When researching the normal distribution of variables, the Shapiro Wilks test was used due to unit counts. Differences between the groups used the Mann Whitney U and Kruskal Wallis H tests due to abnormal distribution of variables. If significant differences were observed with the Kruskal Wallis h test, post-hoc multiple comparison test was used to determine which groups were different. Analyses of more than two dependent variables used the Friedman's two-way ANOVA due to abnormal distribution, with the multiple comparison test used to identify which variable caused the differences if significant differences were present. Chi-square analysis was applied when investigating correlations between groups of nominal variables. In situations where expected values in divisions in 2 x 2 tables had insufficient volume, Fisher's exact test was used and RxC tables had the Pearson chi-square analysis with Monte Carlo simulation applied. When interpreting results, significance value of 0.05 was used with p values less than 0.05 considered to be statistically significant.

## 3. Results

Coronary angiography was performed in all patients included in the study. Stent implantation was performed in all anterior and inferior MI patients, 30 (60%) of NSTEMI patients, and 10 (20%) of high-risk USAP patients.

No significant difference was found for ACS and sex. There were statistically significant correlations found for ACS with complications forming after MI, TVCAD, and mortality status (Table 1). Mortality occurred in 3 patients associated with arrhythmia.

PCT, MCHC, MPV, and Gensini score were not significant according to sex. However, mortality, TVCAD, complications after MI, and ACS were found to be statistically significant (Table 2).

There was no significant difference between age with ACS and subgroups (p>0.05). In terms of PCT values, there were statistically significant differences between ACS groups. Patients with inferior, NSTEMI, and UA diagnosis had PCT values significantly low compared to MI patients (Table 3).

Inferior and anterior subgroups and NSTEMI and UA groups had statistically significant differences between the times for cTnI values. Inferior, anterior, and NSTEMI groups had significantly high cTnI values at 0, 6, and 12 hours compared to the UA group. Additionally, the anterior group had significantly high values for 12-hour cTnI compared to the NSTEMI group. Statistically significant differences were found for the values among the ACS groups (Table 3).

In terms of PCT values, there were statistically significant differences between the ACS groups. PCT level in the anterior MI group was significantly high compared to the inferior MI, NSTEMI, and UA groups (Table 3).

The LVEF value on echocardiography was lowest in the anterior MI group and highest in the UA group, and there were statistically significant differences between the ACS groups (Table 3).

Correlation analysis between PCT, MCHC, and MPV found statistically significant differences (Table 4).

**Figure 1:**
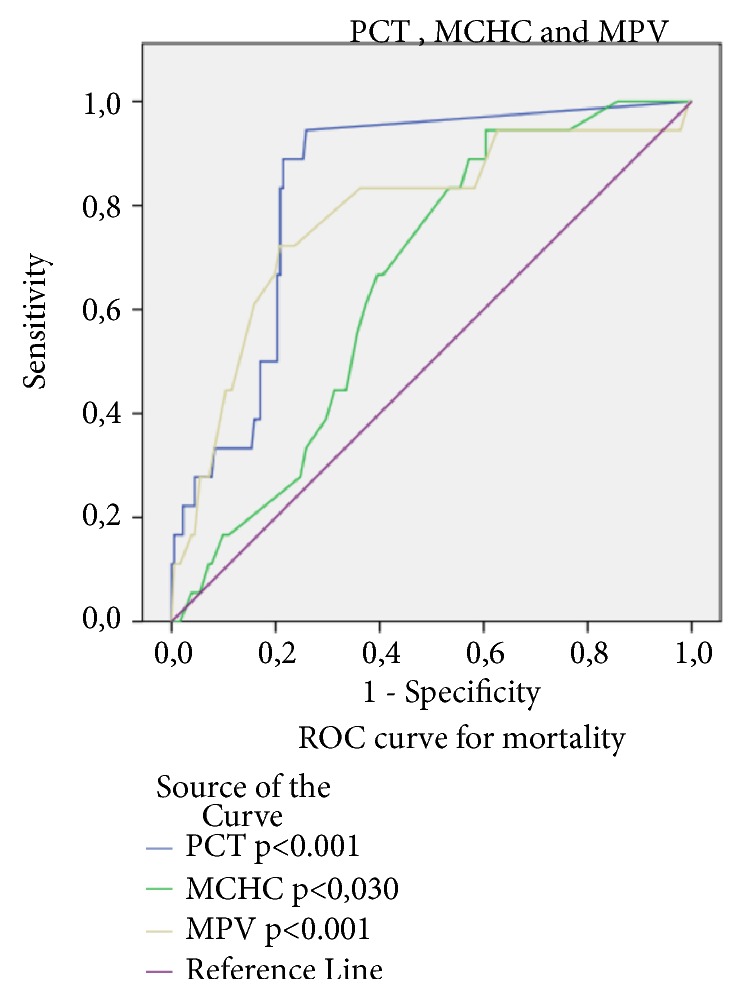
ROC curve analysis according to PCT, MCHC, and MPV mortality positivity.

According to ROC curve analysis, the optimal cut-off values for PCT, MCHC, and MPV to determine positivity for mortality were PCT>0.055 (AUC; 0.834, P=0.001) sensitivity 94.4% and specificity 68.6%; MCHC>34.95 (AUC; 0.656, p=0.030) with sensitivity 83.3% and specificity 57.3% and MPV >9.25 (AUC: 0.779, p=0.001) with sensitivity of 90.1 % and specificity of 62.6% (Figure 1).

**Figure 2:**
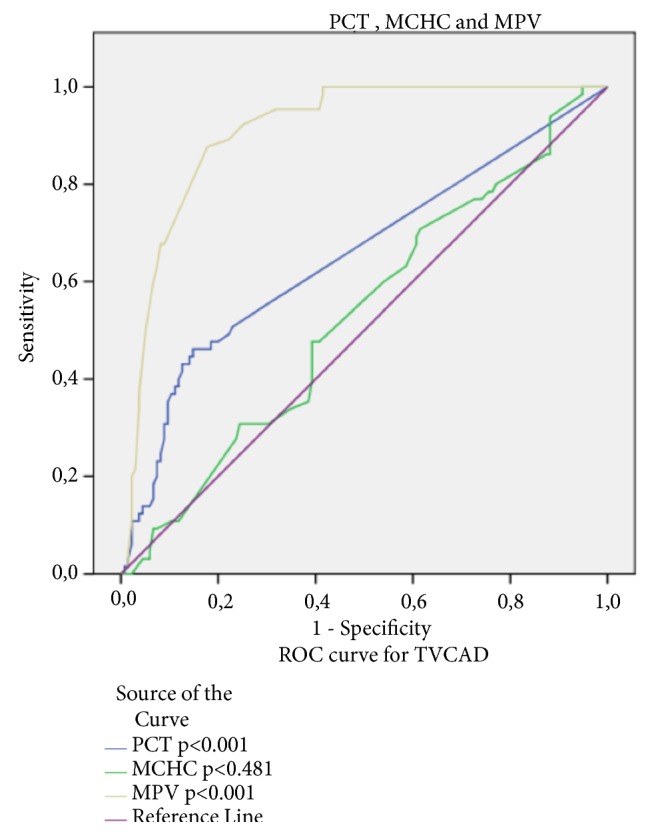
ROC curve analysis according to PCT, MCHC, and MPV TVCAD positivity.

According to ROC curve analysis, the optimal cut-off values for PCT, MCHC, and MPV to determine TVCAD positivity were PCT>0.055 (AUC; 0.654, P=0.001) with sensitivity 78.9% and specificity 61.3%, MCHC>32.85 (AUC;0.531, p=0.481) with sensitivity 69.2% and specificity 56.4% and MPV>8.95 (AUC: 0.908, p=0.001) with sensitivity 87.7% and specificity 69.6% (Figure 2).

## 4. Discussion

To date, we did not identify any study about MCHC, MPV, and PCT together in relation to ACS in the literature. Due to the very low amount of studies about MCHC, MPV, and PCT levels in terms of complications developing after ACS, TVCAD, and mortality rates, we performed our study to identify these correlations.

Anemia is a significant independent risk factor for development of ACS. Anemia reduces the oxygen content available in blood triggering MI via increased myocardial oxygen requirements [16]. A study by Sabatine et al. [17] with 39,922 patients revealed that anemia was an independent and significant risk factor for the occurrence of unwanted cardiovascular events in ACS patients. Studies have found increased recurrent ischemia and death risk in patients with NSTEMI and anemia. A study by Sjauw et al. [18] found each 1 mmol/L (1.61 g/dL) increase in hemoglobin levels caused a nearly 21% reduction in 30-day mortality.

In our study, the presence of anemia in patients was associated with mortality and complications developing after MI. The correlation of MCHC values, Gensini score, and left ventricle ejection fraction (LVEF) in patients and strong positive analysis are important in terms of determining prognosis. Patients with high Gensini score and low LVEF have high mortality rates and poor prognosis. In our study, sensitivity was 83.3% with specificity of 57.3% of MCHC for mortality, while sensitivity was 69.2% and specificity was 56.4% for TVCAD.

A study by Piccini et al. [19] reported that anemic ACS patients had higher death rates linked to ventricular arrhythmia like ventricular tachycardia (VT) and ventricular fibrillation (VF). In our study, 105 (52.5%) of patients did not have any complication observed. VT, acute pulmonary edema, and ischemic heart failure (IHF) were observed to develop in 95 patients (47.5%). The most common of these complications was IHF identified in STEMI patients. Of ACS patients included in the study, 69 (34.5%) had IHF. Of patients, 16 (8%) had VT and 10 (5%) had acute pulmonary edema observed. VT and acute pulmonary edema were identified more frequently in the anterior group.

Platelets are known to play a critical role in pathogenesis of Mi, reblockage of coronary arteries, and revascularization [20, 21]. Additionally, increased MPV is associated with poor prognosis in acute MI [22, 23]. Patients with acute STEMI were shown to have increased MPV [24]. In our study, we identified high correlations between MPV with ACS and subgroups. MPV was identified to have strong positive correlations with ACS and subgroups and also Gensini score and TVCAD with Spearman analysis. We think high Gensini score causes increased poor prognosis and mortality.

Endler et al. [25] in studies comparing patients with stable angina pectoris and acute MI reported MPV levels were higher in patients with acute MI diagnosis. MPV was proposed to be an independent risk factor for ACS. Martin et al. [26] revealed MPV was an independent risk factor for recurrent acute coronary events and mortality after MI.

We identified similar results in our study. MPV rates were high in the ACS groups. Additionally, MPV was found to be closely associated with mortality, TVCAD, and complications developing after AMI. MPV was most frequently high with UA. It was identified that MPV elevation was correlated with PCT and MCHC. Additionally, there were close connections with cTnI. Patients with elevated PCT, MCHC, MPV, and cTnI were correlated with higher complications, TVCAD, and mortality rates. MPV had 90.1% sensitivity and 62.6% specificity for mortality with 88.7% sensitivity and 69.9% specificity for TVCAD.

Puzzili et al. [27] identified higher MPV levels in ACS patients requiring emergency angioplasty compared to those not requiring it. Smyth et al. [28] reported MPV was significantly higher among patients developing restenosis after successful single vein angioplasty and that MPV may be associated with restenosis. In our study, we identified a strong correlation between MPV with TVCAD, Gensini score, and mortality after AMI. There was a negative correlation with ACS and subgroups. This shows that as MPV value increases, the prevalence of coronary artery disease increases and mortality worsens.

The basis of ACS is known to be the inflammatory disease of atherosclerosis. Biasucci et al. [29] proposed that PCT release occurred linked to inflammatory events occurring during AMI and that PCT levels increased in ACS patients. Sinning et al. [30] reported that initially increased PCT levels were associated with increased cardiovascular event rates during follow-up and higher mortality. In our study, in the UA group with lower inflammation compared to the other groups, PCT elevation was identified to be lower compared to the other groups. However, significantly high PCT levels were identified in STEMI and NSTEMI. These findings support the view that PCT may be increased linked to inflammatory events occurring after ACS.

We identified a significant correlation between PCT levels studied in blood samples taken at the time when patients attended with ACS and mortality. At the same time, this correlation was identified for cTnI levels studied at 0, 6, and 12 hours. In accordance with the literature, elevated PCT and cTnI were calculated to be associated with increased mortality in ACS. Additionally, PCT had sensitivity of 94.4% and specificity of 68.6% for mortality.

One of the most frequent complications occurring after MI is IHF. One study showing a correlation between IHF and PCT by Remskar et al. [8] identified PCT levels were high with complications like pulmonary edema and cardiogenic shock. In our study, patients developing IHF linked to STEMI were observed to have higher PCT values compared to UA and NSTEMI patients. When correlations of heart failure development with both cTnI and PCT are evaluated, we identified significant correlations between both high cTnI and high PCT values with heart failure. Especially elevated PCT in heart failure may be an important pathophysiologic factor in inflammation and is considered to affect patient prognosis.

The study by Şentürk et al. identified elevated PCT and cTn levels in those with high TVCAD and that it was simultaneously correlated with mortality [7]. In our study, groups with high PCT and cTn had higher mortality, TVCAD, and complications. Additionally, Gensini score was high and LVEF was low. This shows that PCT may be a predictive value for determination of prognosis like cTn. Also, different to other studies, in our study, ACS was divided into subgroups and we investigated the correlation between PCT, MCHC, and MPV values with anterior and inferior subgroups. Spearman correlation analysis calculated a strong positive correlation between PCT, MCHC, and MPV. Based on this, we identified PCT and MCHC levels in the anterior MI subgroup were significantly high compared to the other subgroups. When we researched the correlation between TVCAD and mortality in the subgroups, we identified significantly higher rates in the inferior MI subgroup. Additionally, Gensini score was higher and LVEF was lower in the anterior subgroup compared to the other groups. The high PCT and MPV rates were observed to follow TVCAD and mortality, while high PCT and MCHC were observed to follow high complications and poor prognosis. Additionally, the sensitivity of PCT was 78.0% and specificity was 61.3% for TVCAD. Based on these findings in our study, contrary to previous studies, we think PCT may be a prognostic factor like troponin and the cheap methods of MCHC and MPV values may have strong effect on prognosis.

In this study, we identified PCT, cTn, MCHC, and MPV levels increased in ACS. We identified that the levels of these four markers increased less in situations with less inflammation like UA compared to other groups. Additionally, similarly in NSTEMI these values increased less compared to STEMI. This leads to the consideration that PCT, cTn, MCHC, and MPV concentrations are correlated with the degree of atherosclerosis in ACS patients and are released linked to inflammatory events occurring during acute MI.

## 5. Conclusion

Increased PCT level may be a useful predictive marker for prognosis in ACS, independent of cTn, along with MCHC and MPV for TVCAD, complications developing, and mortality.

## Figures and Tables

**Table 1:** Chi-Square test result relation between acute coronary syndrome and variables.

		AcuteCoronarySyndrome										Chi-Square Test	
		İnferiorGroup		AnteriorGroup		NSTEMI		UA		Total			
		n	%	n	%	n	%	n	%	n	%	*χ*2	p
Gender	Female	20	40	21	42	19	38	14	28	74	37	2,48	0,477
	Male	30	60	29	58	31	62	36	72	126	63		
	Total	50	100	50	100	50	100	50	100	200	100		

Complication	No	27	54	18	36	23	46	41	82	105	52,5	*27,99*	*0,001*
	VT	3	6	6	12	7	14	0	0	16	8		
	APE	3	6	4	8	2	4	1	2	10	5		
	IHF	17	34	22	44	18	36	8	16	69	34,5		
	Total	50	100	50	100	50	100	50	100	200	100		

Mortality	No	46	92	40	80	47	94	49	98	182	91	*10,99*	*0,012*
	Yes	4	8	10	20	3	6	1	2	18	9		
	Total	50	100	50	100	50	100	50	100	200	100		

TVCAD	No	33	66	22	44	39	78	41	82	135	67,5	*19,94*	*0,001*
	Yes	17	34	28	56	11	22	9	18	65	32,5		
	Total	50	100	50	100	50	100	50	100	200	100		

NSTEMI:Non-ST Elevation Myocardial Infarction, UA: Unstable Angina, VT: Ventricular Tachycardia, APE: Acute Pulmonary Edema, IHF: Ischemic Heart Failure, and TVCAD: Three-Vessel Coronary Artery Disease.

**Table:** (a) Mann-Whitney U test for variance between ACS, gender, mortality, TVCAD, and complications in terms of values

PCT, MCHC, MPV, and GS						
			n	%	Z	P
PCT	Gender	Female Male	*74* *126*	*37* *63*	*-1,077 -0,105* *-0,346* *-0,290*	0,281
MCHC						0,916
MPV						0,729
GS						0,772

PCT	Mortality	No Yes	*182* *18*	*91* *9*	*-6,883 -2,182* *-3,903* *-3,303*	*0,001*
MCHC						*0,029*
MPV						*0,001*
GS						*0,001*

PCT	TVCAD	No Yes	*135* *65*	*67,5* *32,5*	*-4,002 -0,707* *-9,361* *-6,860*	*0,001*
MCHC						0,480
MPV						*0,001*
GS						*0,001*

Total			*200*	*100*		

MCHC: Mean Corpuscular Hemoglobin Concentration, MPV: Mean Platelet Volume, ACS: Acute Coronary Syndrome, GS: Gensini Score, and PCT: Procalcitonin.

**Table:** (b) Regarding values, the relationship between ACS, gender, mortality, TVCAD, and complications Kruskal Wallis H Test Result.

PCT, MCHC, MPV, and GS						
			n	%	H	P
PCT	Complication	No VT APE IHF	*105* *16* *10* *69*	*52,5* *8* *5* *34,5*	*14,748 78,137* *13,238* *26,025*	*0,002*
MCHC						*0,001*
MPV						*0,004*
GS						*0,001*

PCT	Mortality	İnferiorgroup Anteriorgroup NSTEMI AU	*50* *50* *50* *50*	*25* *25* *25* *25*	*11,496 135,189* *31,276* *68,404*	*0,001*
MCHC						*0,009*
MPV						*0,001*
GS						*0,001*

Total			*200*	*100*		

**Table 3:** Kruskal Wallis H Test Result Related to the difference between ACS groups in terms of values.

		Acute Coronary Syndrome						Kruskal Wallis H Test		
		n	Mean	Median	Min	Max	SD	Avarage	H	p
Age	İnferiorGroup	50	64,36	66	42	78	8,33	106,37	1,533	0,675
	AnteriorGroup	50	63,94	64,5	45	97	10,46	101,15		
	NSTEMI	50	63,7	64	37	82	9,24	102,06		
	UA	50	62,12	61,5	33	79	9,74	92,42		
	Total	200	63,53	64	33	97	9,44			

PCT	İnferiorGroup	50	0,29	0,05	0,05	4,84	0,87	168,23	*135,189*	*0,001*
	AnteriorGroup	50	2,08	1,2	0,08	9,98	2,21	83,64		
	NSTEMI	50	0,43	0,05	0,05	9,81	1,57	79,76		
	UA	50	0,07	0,05	0,05	1,03	0,14	70,37		
	Total	200	0,72	0,05	0,05	9,98	1,62	4-1 3-1 2-1		

MCHC	İnferiorGroup	50	34,53	34,8	32	38	1,37	107,81	*11,25*	*0,01*
	AnteriorGroup	50	34,68	35	31	36,8	1,31	118,69		
	NSTEMI	50	34,11	34,2	31,1	37	1,38	92,19		
	UA	50	33,35	34	12	36	3,6	83,31		
	Total	200	34,17	34,4	12	38	2,2	4-1		

MPV	İnferiorGroup	50	8,62	8,35	6,5	13,7	1,45	87,56	*9,052*	*0,029*
	AnteriorGroup	50	8,66	8,6	7,2	16,9	1,37	94,67		
	NSTEMI	50	8,9	8,6	6,1	18,3	1,9	99,22		
	UA	50	9,2	8,85	7,2	16	1,64	120,55		
	Total	200	8,85	8,6	6,1	18,3	1,61	2-4		

LVEF	İnferiorGroup	50	46,7	40,5	28,5	65	11,6	74,41	34,673	*0,001*
	AnteriorGroup	50	43,32	46	20	65	11,81	89,64		
	NSTEMI	50	48,76	49,5	25	65	11,3	98,75		
	UA	50	56,62	60	30	70	9,43	139,2		
	Total	200	48,85	50	28,25	70	12,04	2-4 1-4 3-4		

0.h cTn	İnferiorGroup	50	2,47	2,19	0,02	3,3	2,34	115,67	*113,599*	*0,001*
	AnteriorGroup	50	4,41	2,94	0,04	45	6,59	132,61		
	NSTEMI	50	2,23	1,38	0,05	9,02	2,2	106,07		
	UA	50	0,06	0,04	0	0,8	0,11	27,65		
	Total	200	2,54	1,71	0	45	3,99	4-3 4-2 4-1		

6.h cTn	İnferiorGroup	50	7,41	6,17	1,82	31,3	4,10	123,80	119,428	*0,001*
	AnteriorGroup	50	10,77	9,13	1,07	45,03	8,48	136,31		
	NSTEMI	50	6,79	4,12	0,17	32,4	7,04	105,91		
	UA	50	0,13	0,06	0,01	1,4	0,25	25,86		
	Total	200	6,77	5,67	0,01	45,03	7,3	4-3 4-1 4-2		

12.h cTn	İnferiorGroup	50	23,31	19,91	4,19	63,35	13,43	134,74	*121,067*	*0,001*
	AnteriorGroup	50	27,48	19,28	3,21	100	21,38	137,07		
	NSTEMI	50	15,32	8,92	1,36	56,23	14,48	104,42		
	UA	50	0,34	0,08	0,01	2,24	0,57	25,77		
	Total	200	16,61	10,65	0,01	100	17,79	4-3 4-1 4-2 3-2		

GS	İnferiorGroup	50	59,08	47	6	156	41,24	128,43	*68,404*	*0,001*
	AnteriorGroup	50	62,14	52,5	7	164	41,27	130,5		
	NSTEMI	50	39,48	27	1	142	35,84	96,26		
	UA	50	15,16	9	0	168	25,71	46,81		
	Total	200	43,96	32	0	168	40,88	4-3 4-1 4-2 3-1 3-2		

**Table 4:** Spearman correlation coefficients for PCT, MCHC, and MPV.

	PCT		MCHC		MPV	
	r	p-value	r	p-value	r	p-value
Complication	*0,156*	*<0,001*	*0,606*	*<0,001*	*0,184*	*<0,009*
GS	*0,340*	*<0,001*	*0,202*	*<0,004*	*0,445*	*<0,001*
Mortality	*0,400*	*<0,001*	*0,155*	*<0,029*	*0,277*	*<0,001*
TVCAD	*0,301*	*<0,001*	0,050	>0,481	*0,664*	*<0,001*
ACS	*-0,730*	*<0,001*	*-0,223*	*<0,002*	*-0,218*	*<0,002*
Blood suger	0,138	>0,051	0,120	>0,091	0,085	0,229
LVEF	*-0,305*	*<0,001*	*-0,285*	*<0,010*	0,085	0,229

## Data Availability

The data used to support the findings of this study are available from the corresponding author upon request.
